# Maternal protein restriction induces renal AT2R promoter hypomethylation in salt‐sensitive, hypertensive rats

**DOI:** 10.1002/fsn3.2113

**Published:** 2021-01-27

**Authors:** Moe Miyoshi, Yasuhisa Imakado, Lila Otani, Misa Kaji, Yuki Aanzai, Naoya Sugimoto, Tetsuo Murakami, Masashi Fukuoka, Hirohiko Hohjoh, Huijuan Jia, Hisanori Kato

**Affiliations:** ^1^ Health Nutrition Graduate School of Agricultural and Life Sciences University of Tokyo Tokyo Japan; ^2^ Department of Food Science and Nutrition Faculty of Agriculture Kinki University Nara Japan; ^3^ National Institute of Neuroscience NCNP Tokyo Japan

**Keywords:** angiotensin type 2 receptor, DNA methylation, maternal protein restriction, salt sensitivity, Stroke‐Prone Spontaneously Hypertensive Rat

## Abstract

**Scope:**

We previously demonstrated that protein restriction *in utero* induced salt‐sensitive hypertension and changed renal levels of angiotensin type 2 receptor (AT2R) in Stroke‐Prone Spontaneously Hypertensive Rat (SHRSP). Here, we investigated if this characteristic alteration of AT2R is related to AT2R DNA methylation profiles.

**Methods and Results:**

First, we examined the relation between AT2R DNA methylation and its promoter activity in vitro. Luciferase assays revealed a negative correlation between these two variables. Next, we fed SHRSP dams and grand‐dams a control 20% casein diet or a 9% casein diet during pregnancy. Adult offspring and grand‐offspring were supplied either water or 1% saline solution for 2 weeks. Renal AT2R promoter DNA near the TATA‐box was hypomethylated, mRNA expression was suppressed, and protein expression tended to be higher, in adult offspring of mothers fed a low casein diet. Moreover, adult grand‐offspring exhibited high blood pressure after salt loading, along with suppressed transcription of AT2R mRNA and elevated translated protein.

**Conclusions:**

Under a fetal environment of protein restriction, the increase in protein expression due to hypomethylation of the AT2R promoter region occurs as a response to increased salt sensitivity, and controlling this mechanism may be important for the prevention of hypertension.

## Graphical abstract

Offspring and grand‐offspring derived from rats fed low‐protein diets during pregnancy showed significantly increased blood pressure upon drinking saline solution (increasing the risk of salt‐sensitive hypertension). As a potential molecular mechanism, we found that the AT2R level that regulates blood pressure is increased through DNA hypomethylation in the promoter region. Controlling this characteristic change observed in response to salt loading could help prevention and treatment of hypertension.

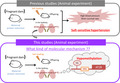

## 1 INTRODUCTION

Hypertension is one of the strongest risk factors for cardiovascular diseases (Lim et al., 2012). In most cases, patients are diagnosed with essential hypertension that has no identifiable cause. In addition to genetics, environmental factors such as high salt intake, alcohol consumption, and a sedentary lifestyle are thought to increase the risk of developing essential hypertension (Cutler et al., 1997; Dickinson et al., 2006; Singh et al., 2016; Whelton et al., 2002; Xin et al., 2001) Not only lifestyle of the individual but also fetal environment is an important factor for hypertension development. For example, numerous studies have proven that maternal protein restriction is also closely related to onset of hypertension (Brennan et al., 2006; Zohdi et al., 2014).

We previously demonstrated that maternal protein restriction and subsequent offspring's salt intake induced salt sensitive hypertension and shortened life span in the offspring of the Stroke‐Prone Spontaneously Hypertensive Rat (SHRSP) (Otani et al., 2004), which is generally regarded as a useful genetic model of hypertension and stroke (Trippodo & Frohlich, 1981). In order to clarify the molecular mechanism of salt sensitive hypertension, we conducted a study focusing on the renin‐angiotensin aldosterone system which plays a pivotal role in blood pressure regulation. Interestingly, we revealed that the offspring exposed to maternal protein restriction and salt loading to offspring had significantly higher levels of renal angiotensin type 2 receptor (AT2R) whereas angiotensin type 1 receptor (AT1R) expression and angiotensin‐converting enzyme (ACE) activity did not differ (Otani et al., 2012).

Renal AT2R mainly functions in vasodilation and tissue protection; when activated in SHRSP rats, AT2R improves survival rate and renal damage (Brouwers et al., 2013; Gelosa et al., 2009). The importance of AT2R and AT2R‐mediated signaling has also been suggested for the molecular mechanism of salt‐sensitive hypertension (Hong & Garvin, 2012). However, we still do not fully understand how maternal protein restriction and salt loading regulate AT2R protein profiles.

Several epidemiological studies and animal experiments reported that maternal protein restriction is associated with increased hypertension in later life. This link occurs because environmental stress alters the epigenome, particularly DNA methylation profiles (Bogdarina et al., 2007; Heijmans et al., 2008). We previously reported that exposing SHRSP fetuses to protein restriction accompanied by altered methylation and elevated gene expression of prostaglandin E Receptor 1 (*Ptger1*), one of the key mediators of sodium retention (Miyoshi et al., 2018) Additionally, perinatal nicotine exposure altered site‐specific AT2R promoter CpG methylation and repressed its gene expression in the brain, increasing brain hypoxic‐ischemic injury in neonatal rats (Li et al., 2012, 2013). Thus, we hypothesized that the characteristic AT2R protein expression observed in our previous study was due to altered AT2R methylation. To test this hypothesis, here we used renal cells in vitro to investigate whether a correlation exists between DNA methylation and its promoter activity. Next, we evaluated renal AT2R methylation profiles using SHRSP offspring exposed to maternal protein restriction *in utero*. In addition, as for AT2R protein level and its mRNA expression, we also examined using grand‐offspring derived from protein restricted grand‐dams during pregnancy.

## 2 EXPERIMENTAL SECTION

### 2.1 Construction of reporter plasmids and partial methylation

The predicted AT2R promoter region (positions −2816 to + 77 from AT2R transcription start site) was amplified using a LongAmp Taq DNA Polymerase (New England Biolabs), rat genomic DNA as a template, and PCR primers (sense: 5ʹ‐GAAAGAATAGGTTCTCAGAAAAATG‐3ʹ; antisense: 5ʹ‐TTCAGATCTTCCGAGTCAAGCATGTGTA‐3ʹ). Amplicons and pGL3‐luciferase reporter (basic; Promega) were digested with BglII and NheI (New England Biolabs) and ligated. Partial methylation was performed as according to Tamura *et al*. (2007). Methylated DNA levels were determined via digestion with SmaI and XbaI (New England Biolabs), followed by electrophoresis.

### 2.2 Cell culture and transfection

Human embryonic kidney 293t cells were cultured in Dulbecco's modified eagle medium (DMEM; Sigma‐Aldrich) supplemented with 10% fetal bovine serum and 1% penicillin‐streptomycin (Thermo Fisher Scientific). Cells were incubated at 37°C in 5% CO_2_. For transfection, cells were seeded on 24‐well tissue culture plates until 80% confluence (approximately 1.25 × 10^5^ cells/well). On transfection day, growth medium was removed and replaced for 1 hr with DMEM lacking antibiotics. Cotransfection of constructed luciferase reporter plasmid (0.15 µg) and control phRL‐TK plasmid (0.05 µg) (Promega) was performed using Lipofectamine 3,000 in Opti‐MEM™ medium (Thermo Fisher Scientific, Invitrogen) following manufacturer protocol. Cells were incubated at 37°C and 5% CO_2_ for 24 hr to prepare cell lysate. Luciferase activity was examined using the Dual‐Luciferase Reporter Assay System (Promega) following manufacturer protocol.

### 2.3 Animals

The present study was approved and conducted in strict accordance with the guidelines stipulated by “The Animal Usage Committee of the Graduate School of Agricultural and Life Sciences” at the University of Tokyo (Approval No. P09‐376). Rats were maintained at 22 ± 1°C in 60% ± 5% humidity and in a 12 hr light (8:00 to 20:00)/dark cycle.

Animal experiments followed previously published procedures (Supporting Information Figure S1) (Otani et al., 2012). The animals were bred with sibling SHRSP. Virgin female SHRSP weighing 150–180 g were mated to the same strain male (weighing 176 g or more, blood pressure 180–185 mmHg). F0 animals were paired with one male to one female and confirmation of a vaginal plug was set as pregnant day 0. F0 animals were provided pair feeding and given a 20% and a 9% casein diet in the control and low‐protein groups, respectively, from pregnant day 0 to the confirmation of delivery (Table S1). The feed was substituted by a commercial diet (Funabashi SP, Funabashi Farm Co.) after the confirmation of delivery. F1 rats were culled on postnatal day 4, resulting in a uniform litter size of 6. All F1 rats were provided commercial diet after weaning. We previously reported that 9% casein diet feeding were not altered blood pressure in SHRSP dams. Blood pressure elevated same levels in the F1 offspring that had been exposed to 20% casein (male, *n* = 16; 190 ± 7 mmHg, female, *n* = 8; 170 ± 11mmHg) and in the F1 offspring that had been exposed to 9% casein (male, *n* = 14; 186 ± 11mmHg, female, *n* = 7; 179 ± 10 mmHg) at the 9 weeks of age.

Several female F1 rats were mated with sibling male at 9 weeks of age, and divided into 3 groups: (a) grand‐dam SHRSP rats (F0) during pregnancy were fed a 20% casein diet, and then pregnant female SHRSP rats (F1) were fed a 20% casein diet (CON group), (b) grand‐dam SHRSP rats (F0) during pregnancy were fed a 20% casein diet, and then pregnant female SHRSP rats (F1) were fed a 9% casein diet (maternal protein restriction, MPR group), (c) grand‐dam SHRSP rats (F0) during pregnancy were fed a 9% casein diet, and then pregnant female SHRSP rats (F1) were fed a 20% casein diet (grand‐maternal protein restriction, GPR group). Dams during lactation and male pups (F2) after birth were reared on a commercial diet (Funabashi SP; Funabashi Farm Co., Ltd.) and water ad libitum. From 10 to 12 weeks old, F2 rats were given water (W group) or 1% saline solution (salt loading, S group); control rats were also split into W and S groups. At 12 weeks, offspring were euthanized for kidney collection.

### 2.4 Blood pressure measurement

Blood pressure of 12‐week‐old male offspring was measured as previously described (Otani et al., 2012), using the tail‐cuff method (BP‐98A; Softron).

### 2.5 Measurement of relative DNA methylation

Total DNA was isolated from kidney tissue using a phenol‐chloroform‐isoamyl alcohol (25:24:1) mixture (Sigma‐Aldrich), then resuspended in 400 μl of Tris‐HCl buffer (pH 8.0; NIPPON GENE). Next, it was bisulfite‐converted using an EZ DNA Methylation‐Gold Kit^TM^ (Zymo Research) and amplified using BioTaq DNA Polymerase (Bioline) with *Agtr2* primers (sense, 5ʹ‐GGGTATTATATGGAATTTTATTTTTGT‐3ʹ; antisense, 5ʹ‐ACTACACCAAACCTCTAATTTCCTTC‐3ʹ). These template PCR products were ligated into the pGEM‐T easy vector (Promega) and transformed into competent *Escherichia coli* DH5α cells (Sigma‐Aldrich), generating cloning sequence libraries. Plasmids containing target DNA were extracted using the GenElute^TM^ Plasmid MiniPrep Kit (Sigma‐Aldrich); clones were then commercially sequenced by Eurofins Genomics. Obtained data was analyzed with the Quantification Tool for Methylation Analysis (RIKEN Center for Developmental Biology).

### 2.6 Protein extraction and Western blots

Procedures followed previous publications (Otani et al., 2012). Briefly, protein samples were extracted from kidney tissues. Primary and secondary antibodies were AT2R polyclonal antibody (1:100, C‐18; Santa Cruz Biotechnology) and horseradish peroxidase (1:2000; BIO‐RAD Laboratories).

### 2.7 RNA extraction and RT‐digital PCR

Total RNA was extracted from kidney samples using TRIzol reagent (Thermo Fisher Scientific) and reverse‐transcribed to cDNA using the PrimeScriptTM RT reagent kit (Takara Bio, Tokyo, Japan). Expression of mRNA was measured on the QuantStudio™ 3D Digital PCR System with 20K Chips v2 (Thermo Fisher Scientific). Reaction mixtures comprised digital PCR (dPCR) Master Mix (Thermo Fisher Scientific), TaqManTM Gene Expression Assays (angiotensin type 2 receptor, *Agtr2*, Rn00560677_s1, FAM‐MGB; Glucuronidase beta, *Gusb*, Rn00566655_m1, VIC‐MGB; Thermo Fisher Scientific), and cDNA. The thermocycling protocol was as follows: 10 min at 96°C, 39 cycles of 2 min at 58°C and 30 s at 98°C, followed by 2 min at 58°C, before maintenance at 10°C. Data were analyzed in QuantStudio™ 3D AnalysisSuite™ Cloud (Thermo Fisher Scientific). The normalization gene was *Gusb*, and relative *Agtr2* mRNA expression was calculated as fold‐change.

### 2.8 Statistical analysis

Data are represented as means ± *SEM*. Determination of relative DNA methylation was performed in triplicate (three randomly selected offspring per group). Between‐group differences in animal experiments were assessed with a one‐way analysis of variance (ANOVA) followed by the Tukey's test. Significance was defined as *p* < .05.

## 3 RESULTS

### 3.1 Correlation between AT2R DNA methylation and expression

**FIGURE 1:**
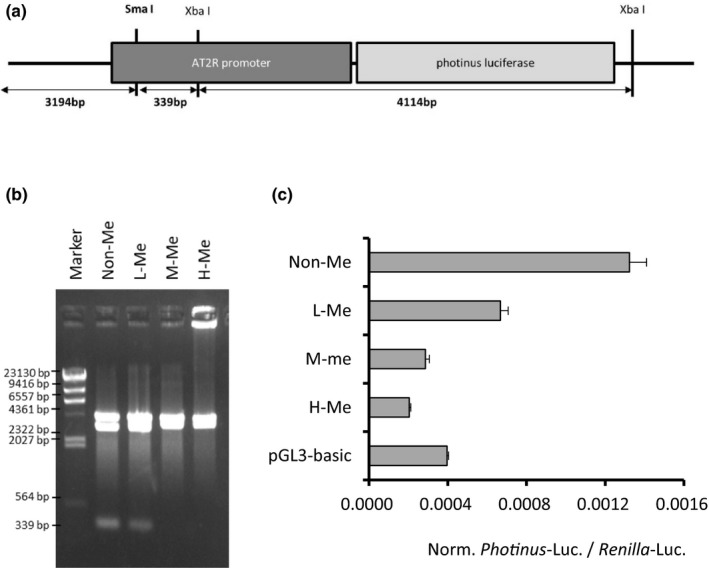
Negative correlation between AT2R DNA methylation and expression levels. (a) Schematic drawing of constructed reporter plasmid, indicating restriction enzyme sites of SmaI and XbaI. (b) Photo of agarose gel electrophoresis. Reporter plasmids with different DNA methylation levels were digested with SmaI and XbaI, then examined using agarose gel electrophoresis. Non‐Me, non‐methylated plasmid; L‐Me, low‐methylated plasmid; M‐Me, moderately methylated plasmid; and H‐Me, highly methylated plasmid. Marker is a HindIII‐digested λ DNA. (c) *Photinus* luciferase activity of reporter plasmids, normalized against *Renilla* luciferase activity as a control. Data represent means ± *SEM* (*n* = 3)

We constructed a reporter plasmid encoding a putative AT2R promoter region linked to the *Photinus luciferase* gene. The AT2R promoter region has a SmaI restriction enzyme site (Figure 1a). Because SmaI cleaves the unmethylated but not the methylated restriction site, degree of cleavage reflects methylation state at the SmaI site and neighboring regions. Results of agarose gel electrophoresis on SmaI‐digested, methylated reporter plasmid confirmed differing levels of DNA methylation (Figure 1b and S2). After methylated reporter plasmids were introduced into cells, *Photinus luciferase* activity decreased with increasing DNA methylation of the AT2R promoter (Figure 1c).

### 3.2 Blood pressure of SHRSP offspring and grand‐offspring

**FIGURE 2:**
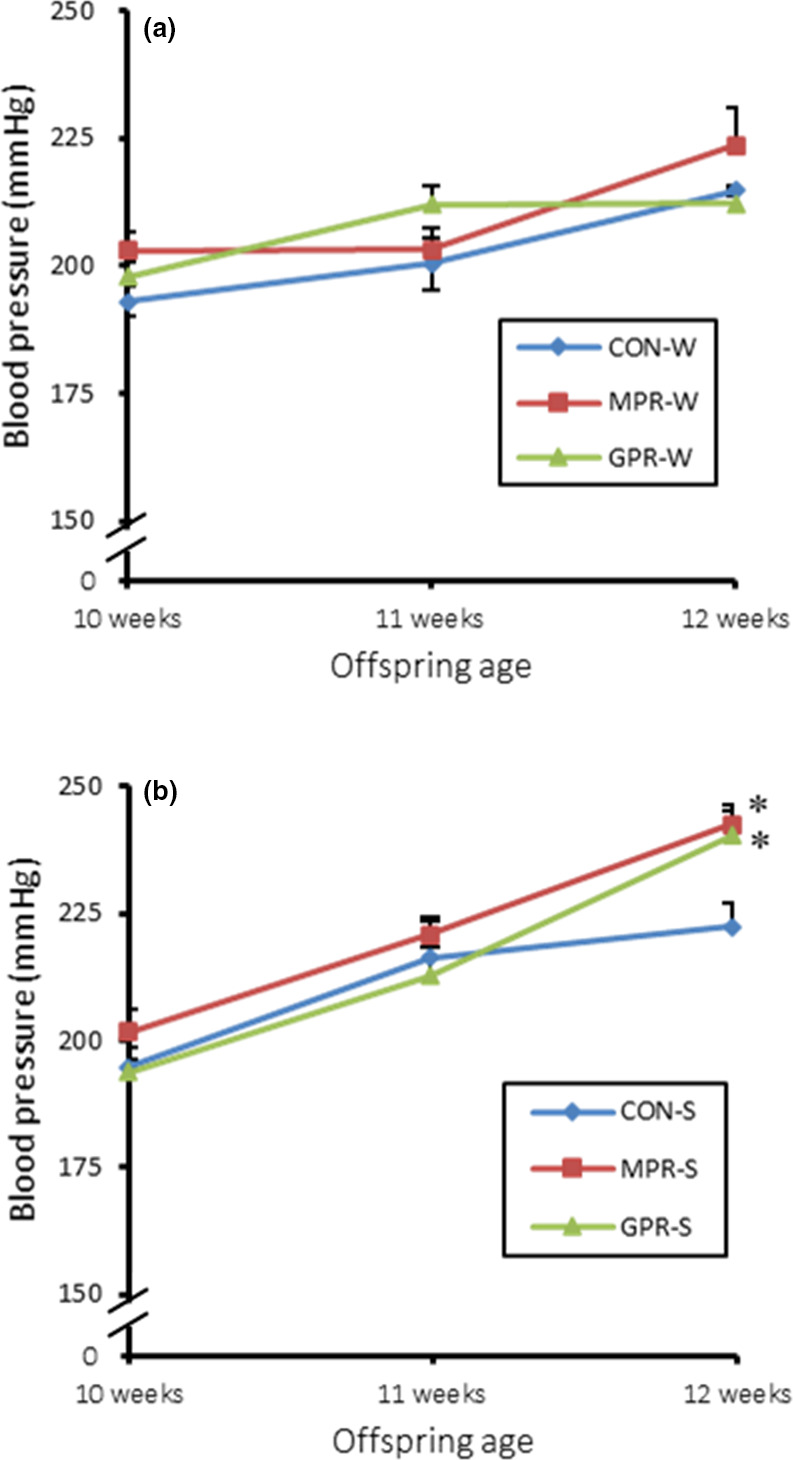
Effect of maternal and grand‐maternal protein restriction on blood pressure. (a) Blood pressure of 12‐week‐old offspring in the water drinking group. (b) Blood pressure of 12‐week‐old offspring in the salt loading group. Values are means ± *SEM* (*n* = 5–7). *p < .05 versus. control, one‐way ANOVA and Tukey's test. Offspring and grand‐offspring were exposed to either a control 20% casein diet or a 9% casein diet in *utero*. In adulthood, they were provided with either water (W) or 1% saline (S) to drink. CON, control; MPR, maternal protein restriction; GPR, grand‐maternal protein restriction; W, water; S, saline

There were no significant differences in blood pressure of 10‐week‐old in the water drinking group and the salt loading group, respectively (Figure 2a, b). In response to salt loading, blood pressure of 12‐week‐old MPR‐S and GPR‐S was significantly increased compared with control (Figure 2b) Under the water drinking, blood pressure of 12‐week‐old did not differ significantly in control, MPR‐W, and GPR‐W (Figure 2a).

### 3.3 Renal DNA methylation profile in AT2R promoter region of SHRSP offspring

**FIGURE 3:**
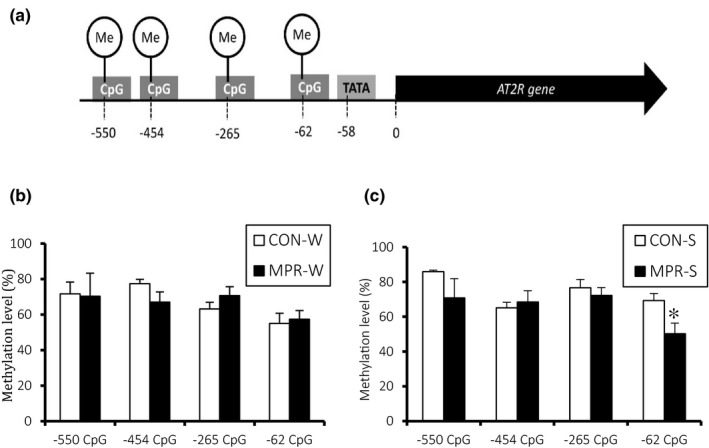
Renal DNA methylation profiles in AT2R promoter region. (a) Schematic profiles of AT2R promoter region. Renal AT2R DNA methylation profiles of the water drinking (b) and salt loading (c) groups. Values are means ± *SEM* (*n* = 3). *p < .05, one‐way ANOVA and Tukey's test. Offspring were exposed either a 20% (CON) or 9% (MPR)‐casein diet *in utero*, and provided with either water (W) or 1% saline (S) to drink in adulthood

We evaluated four CpG sites in the AT2R promoter region (Figure 3a) and did not observe significant differences in methylation profiles among water drinking groups (Figure 3b). Methylation decreased significantly at −62 of MPR‐S compared with control (Figure 3c).

### 3.4 AT2R levels of SHRSP offspring and grand‐offspring

**FIGURE 4:**
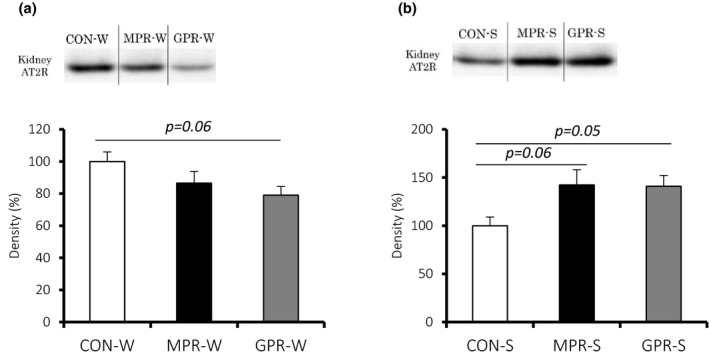
Effect of maternal and grand‐maternal protein restriction on AT2R protein level. Renal AT2R protein levels in the water drinking (a) and salt loading (b) groups. Values are means ± *SEM* (*n* = 5–7). Offspring and grand‐offspring were exposed either a 20% (CON) or 9% (MPR, GPR) casein diet *in utero*, and provided with either water (W) or 1% saline (S) to drink during adulthood

**FIGURE 5:**
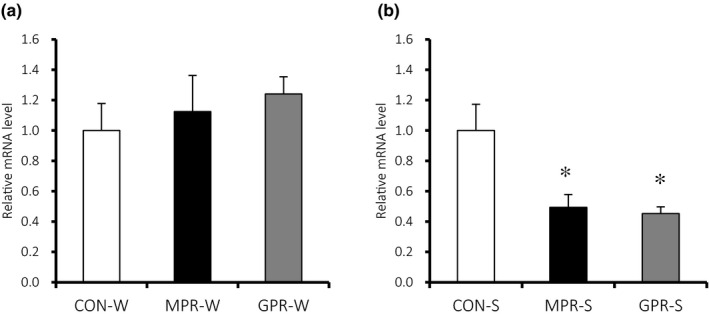
AT2R mRNA expression measured using digital PCR. (a) Water drinking groups. (b) Salt loading groups. *p < .05 versus. CON‐S, one‐way ANOVA and Tukey's test. Values are means ± *SEM* (*n* = 5–7). Offspring and grand‐offspring were exposed either a 20% (CON) or 9% (MPR, GPR) casein diets *in utero*, and provided with either water (W) or 1% saline (S) to drink in adulthood

The kidneys of GPR‐W had lower AT2R protein content than the water‐drinking control (Figure 4a), whereas both MPR‐S and GPR‐S kidneys had more AT2R than their control (Figure 4b). Water drinking groups did not differ significantly in *Agtr2* mRNA levels. However, *Agtr2* mRNA was significantly lower in MPR‐S and GPR‐S than in control (Figure 5a, b).

## 4 DISCUSSION

Here, we demonstrated that methylation of the AT2R promoter region negatively regulates promoter activity in vitro. Furthermore, our experiments using SHRSP rats showed that maternal protein restriction induced a negative correlation between renal AT2R promoter hypomethylation and its protein expression, triggered possibly by the secondary factor of salt loading in adulthood. These data suggest that epigenetic regulation of AT2R is a major cause of the altered AT2R protein expression observed in rats with salt sensitivity in this study and in previous studies (Otani et al., 2012).

Angiotensin II is generated by ACE and acts through two major receptors: AT1R and AT2R. Takeda *et al*. (2001)*,* reported that high salt intake increases aldosterone production and expression of the AT1R mRNA in the cardiovascular tissue in SHRSP, which may contribute to the development of salt sensitivity in SHRSP. Maternal protein restriction also could be associated with decreased renal expression of AT1R and AT2R in offspring and resulting in the inability of renal tubules to handle the hydro‐electrolyte balance, consequently causing arterial hypertension (Mesquita et al., 2010). We also compared the renal expression of AT1R and AT2R, and found a significantly increased protein level of AT2R, but unchanged AT1R protein level and activity of ACE in SHRSP offspring of protein‐restricted dams (Otani et al., 2012).

We first examined the effect of methylation on AT2R promoter activity in kidney cells. As a major epigenetic mechanism of gene regulation, DNA methylation usually occurs at cytosine residues of CpG dinucleotide sequences (Jaenisch & Bird, 2003). In promoter regions, methylation generally inhibits transcription and causes long‐term suppression of target genes (Jaenisch & Bird, 2003). In the current study, the luciferase assay results revealed that DNA methylation of the AT2R promoter negatively regulated protein expression, suggesting that this epigenetic mechanism plays a major role in altering AT2R protein levels.

Our in vivo experiments using SHRSP rats confirmed the in vitro results and corroborated previous studies (Miyoshi et al., 2018; Otani et al., 2004, 2012). Specifically, SHRSP offspring exposed to a low‐protein fetal environment and salt loading in adulthood showed significantly elevated blood pressure as well as altered renal AT2R protein levels. For the first time, we also demonstrated that subjecting grand‐dams to these stressors may have the same epigenetic effects in their grand‐offspring. These results suggest that the increment of salt sensitivity caused by maternal and grand‐maternal protein restriction are passed on from generation to generation.

Salt loading appeared to significantly decrease methylation at the −62 CpG position when fetuses were exposed to low protein levels, suggesting that hypomethylation brings to an increasing AT2R protein expression. Indeed, this hypomethylated region is located four bases upstream of the TATA‐box promoter; elevated methylation in this region suppresses gene expression (Kitazawa & Kitazawa, 2007). This mechanism has been reported for the AT2R promoter region; CpG methylation upstream of the TATA‐box inhibited TBP binding affinity, negatively regulating both gene and protein expression (Li et al., 2012, 2013). AT2R is involved in the development of renal vascular and tubular structures, such as urogenital morphogenesis, vasculogenesis, and vascular differentiation, and is essential for normal kidney formation (Chow & Allen, 2016; Oshima et al., 2001; Yosypiv, 2008). Since the amount of AT2R protein is increased by epigenetic control to antagonize the increase in blood pressure caused by low levels of fetal protein, controlling and enhancing this effect may be effective for the prevention and improvement of salt sensitivity.

In post‐weaning water drinking conditions, due to the effects of maternal protein restriction, the level of AT2R protein tends to decrease and we revealed that this alteration in protein expression is not controlled by its retention as an epigenetic marker. Thus, we found that epigenetic control in AT2R is triggered only if there is a primary factor of low fetal protein exposure and a secondary factor of salt load.

AT2R mRNA expression in the kidneys is relatively high in the fetal period and decreases after birth (Sampson et al., 2012). Thus, in our preliminary experiments, we were unable to detect AT2R mRNA expression in adult male SHRSP rats, even with real‐time PCR. Therefore, we chose dPCR to examine AT2R mRNA expression, as this method can detect very small amount of transcripts for higher accuracy, sensitivity and absolute quantification (Quan et al., 2018). Interestingly, our dPCR results were inconsistent with the hypothesis that AT2R promoter is negatively regulated through hypomethylation, because AT2R mRNA exhibited an opposite pattern when compared with the normal protein diet group. Using dPCR, we were able to demonstrate that, when SHRSP offspring and grand‐offspring were exposed to a low‐protein fetal environment followed by salt loading in adulthood, AT2R mRNA expression decreased significantly compared with the control.

Several studies indirectly support our data. One previous report showed that while AT2R mRNA expression decreases with brain development, AT2R protein levels actually increase (Gao et al., 2012). The same incongruence between AT2R mRNA and protein expression also occurs in the kidneys (Sampson et al., 2012; Gao et al., 2012). Because numerous processes separate transcription and translation, gene and protein expression are often not proportional. In addition to the amount of mRNA transcription, mRNA stability and translation efficiency also control protein levels (Vogel & Marcotte, 2012). Moreover, mechanisms other than epigenetics could regulate AT2R mRNA. Upstream open reading frames and non‐coding RNAs such as micro‐RNAs can regulate transcription (Barrett et al., 2012; Wethmar et al., 2010). Additionally, both poly (ADP‐ribose) polymerase‐1 and NO stress have been shown to control AT2R gene expression (Carey, 2017; Dao et al., 2016; Reinemund et al., 2009) .

Our study has several limitations. First, AT2R receptor expression and function differ greatly depending on the animal model, sex, age, and presence or absence of complications such as obesity (Chow & Allen, 2016; Hilliard et al., 2014; Oshima et al., 2001; Padia et al., 2009; Sampson et al., 2012; Song et al., 2010). Second, we examined the whole kidney, including regions that do not express AT2R (Miyata et al., 1999). Since our research focused on describing the phenomenon in animal models with high risk of hypertension, which we established in the previous research, priority was given to testing under the same conditions as in the previous research, rather than these factors.

dPCR is a useful and powerful tool to compensate for the weaknesses of conventional gene evaluation methods such as real‐time PCR. dPCR is expected to be particularly useful for accurately evaluating the expression levels of target genes that fluctuate in response to nutritional conditions, including conditions of exceptionally low expression levels and/or slight differences among groups.

AT2R had been often noted as a receptor that antagonizes the effects of AT1R‐mediated blood pressure elevation signaling. However, recent studies have suggested that AT2R activation alone may be sufficient to prevent from salt sensitivity without the concomitant addition of AT1R blockade (Ali et al., 2013). It was also reported that SHRRSP given high salt diet with a AT2R agonist shown to be significantly prolonged survival without affecting blood pressure (Gelosa et al., 2009). Our previous research has addressed that SHRSP offspring exposed to maternal protein restriction and post‐weaning salt loading were not affected to AT1R (Otani et al., 2012), and in this study we confirmed that AT2R receptors were increased through DNA hypomethylation. Thus, it is necessary to investigate the suppressive effects on salt sensitivity via activating AT2R by administering an agonist in the future.

In conclusion, we demonstrated that DNA methylation of the AT2R promoter region negatively regulated AT2R protein levels in offspring exposed to maternal protein restriction and post‐weaning salt loading; this effect was not seen in water drinking conditions. This epigenetic control difference in AT2R will be an important perspective for prevention and treatment strategies of hypertension.

## CONFLICTS OF INTEREST

The corresponding author belongs to a university‐community relations laboratory that is associated with Ajinomoto Co., Inc.

## AUTHOR CONTRIBUTIONS

M.M. and H.H. constructed reporter plasmids, in addition to performing methylation, cell culture, and transfection experiments. L.O., M.K., Y.A., N.S., and T.M. conducted animal breeding, blood pressure measurements, and western blots. Y.I. performed the methylome analysis. M.M., M.F., and H.H. performed dPCR. M.M. performed all statistical analyses. M.M wrote the paper. H.H., H.K., and H.J. performed revisions. All authors reviewed the manuscript.

## Supporting information

Supplementary Material
