# Supplementary material for: Maternal protein restriction induces renal AT2R promoter hypomethylation in salt‐sensitive, hypertensive rats
Source: Food Sci Nutr. 2021 Jan 27;9(3):1452–9. doi: 10.1002/fsn3.2113 (PMC7958563; doi:10.1002/fsn3.2113)
Supplement: Supplementary file 1 — Supplementary Material [file FSN3-9-1452-s001.docx]

**Supplementary information**

**Table S1. Composition of synthetic diet**


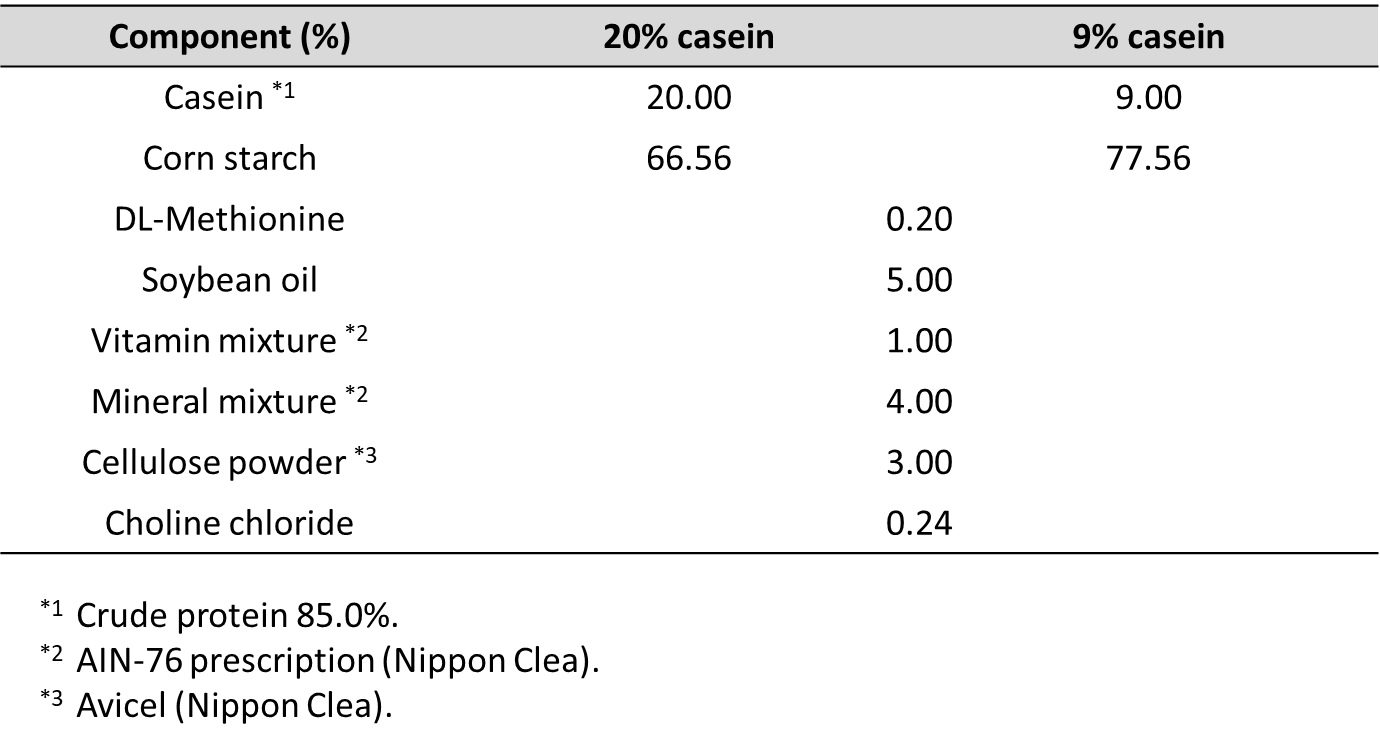


**
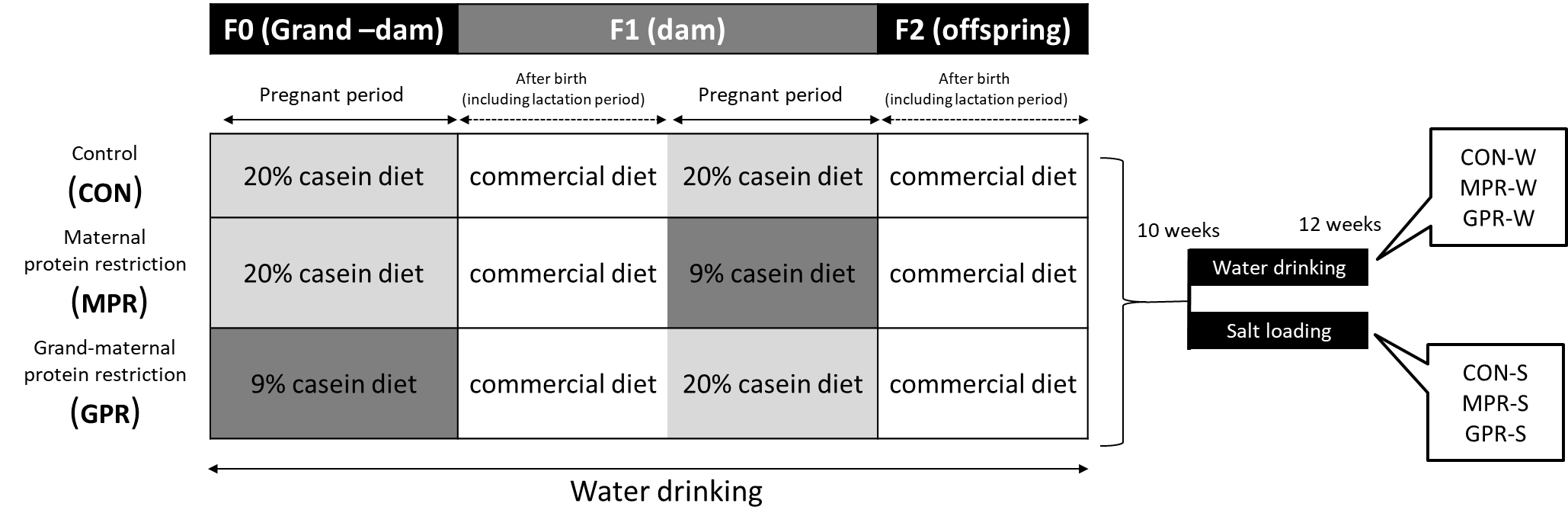
**

**Figure S1. Schematic of animal experiments.**

SHRSP dams and grand-dams were fed 20% casein diet or 9% casein diet while pregnant. After birth, male offspring were given water or 1% saline solution from 10 to 12 weeks old.

| **A　　　　　　　　　　　　　　　　　　B**  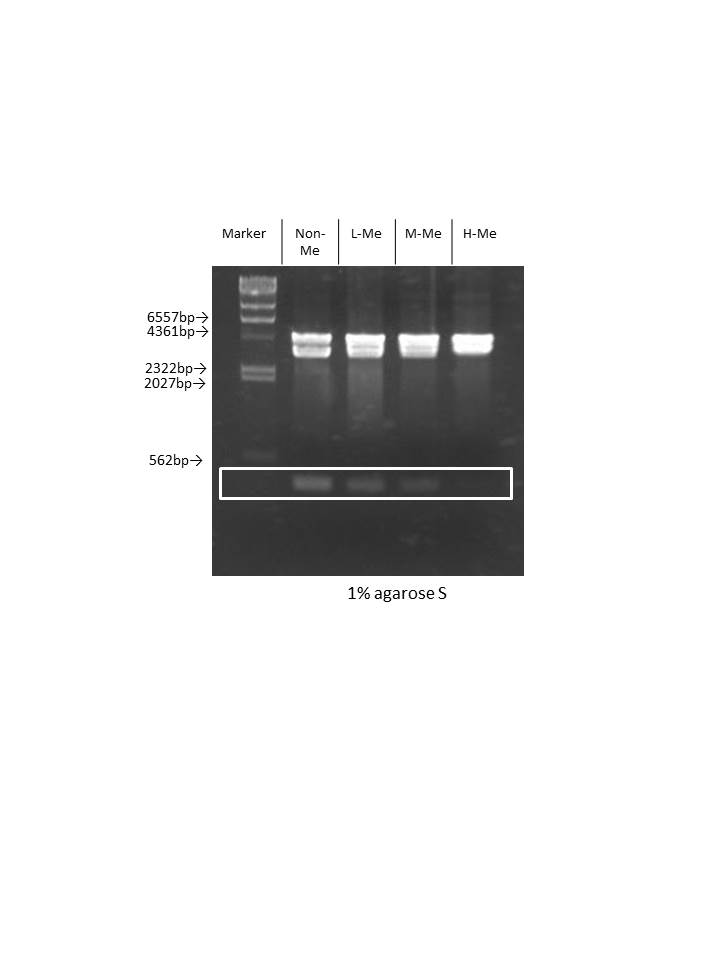 |
| --- |

**Figure S2. Partial methylation of AT2R promoter.**

(A) Results of electrophoresis confirming partial DNA methylation. The size marker was HindIII-cut l DNA. Four different plasmid methylation levels were prepared: non-methylated (Non-Me), lowly methylated (L-Me), moderately methylated (M-Me), highly methylated (H-Me). (B) Area Under the Curve (AUC) of putative 339 bp band, detected using CS Analyzer 3.0 (ATTO, Tokyo, Japan).
